# Mental Misrepresentation in Non-human Psychopathology

**DOI:** 10.1007/s12304-017-9299-2

**Published:** 2017-08-04

**Authors:** Krystyna Bielecka, Mira Marcinów

**Affiliations:** 1 Institute of Philosophy, University of Warsaw, ul. Krakowskie Przedmieście 3, 00-097 Warszawa, Poland; 2 Institute of Philosophy and Sociology, Polish Academy of Sciences, ul. Nowy Świat 72, 00-330 Warszawa, Poland

**Keywords:** Mental misrepresentation, Mental representation, Mental disorder, Dynamical function, etiological function, OCD, Delusion

## Abstract

In this paper, we defend a representational approach to at least some kinds of non-human psychopathology. Mentally-ill non-human minds, in particular in delusions, obsessive-compulsive disorders and similar cognitive states, are traditionally understood in purely behavioral terms. In contrast, we argue that non-human mental psychopathology should be at least sometimes not only ascribed contentful mental representation but also understood as really having these states. To defend this view, we appeal to the interactivist account of mental representation, which is a kind of a constructive approach to meaning. We follow Mark Bickhard in assuming that only an organism – either human or non-human – capable of detecting its own misrepresentations is representational. However, under his autonomy-based account of biological function these minds are incapable of misrepresentations because these minds are, *ex hypothesi*, unable to detect error in such representations. To solve this problem, we argue that adding a historical dimension – as in Millikan’s view on mental representations – to Bickhard’s account of function makes mental misrepresentation of mentally-ill minds possible. Using Bickhard’s dynamic account of function, it is possible to explain why delusions and other mental disorders can be seen as locally functional. However, an etiological dimension can further explain why misrepresentations seem to be globally dysfunctional. Even if representational or biosemiotic hypotheses about non-human psychopathology are difficult to confirm empirically, we defend the view that they can enrich our understanding of the causes and development of such pathologies, and may constitute a new progressive research programme.

In this paper, we propose a constructive approach to mental misrepresentation in non-human psychopathology. By the *constructive approach to meaning* biosemioticians mean an approach according to which the meaning is created in an organism as opposed to one that is merely ascribed by the researcher. The same approach to representation can be found in Bickhard’s account of mental representation as well as in Froese’s development of Varela’s (Maturana and Varela 1980) notion of *autopoiesis* (Froese and Stewart 2010) or in Deacon's (2012) theory of ententional phenomena. In this paper, we restrict ourselves to the teleosemantical account of mental representation. Especially, we focus on Bickhard's (1993, 2008) account because it constitutes a clear attempt to show how an organism can detect the adequacy of its mental representations (by having a capacity for system-detectable error) that – as we will argue – makes representations and misrepresentation really possible in cognitive systems. We will also use Millikan’s (1984, 2005) concept of biological function in order to better elucidate the possibility to misrepresent in mentally-ill minds.

Our argument goes as follows: mentally-ill non-human minds, in particular in delusions, obsessive-compulsive disorders and similar cognitive states, as we show (section 1), are traditionally understood in purely behavioral terms. We argue that non-human psychopathology should be at least sometimes not only ascribed contentful mental representation but also understood as really involving these states. To defend this view, we appeal to the interactivist account of mental representation. We follow Bickhard in assuming that only an organism – either human or non-human – capable of detecting its own misrepresentations is representational (section 2). However, under his autonomy-based account of biological function these minds are incapable of misrepresentations because these minds are, *ex hypothesi*, unable to detect error in such representations (section 3). To solve this problem, we argue that adding a historical dimension – as in Millikan’s view on mental representations – to Bickhard’s account of function makes mental misrepresentation of mentally-ill minds possible. Using Bickhard’s dynamic account of function, it is possible to explain why delusions and other mental disorders can be seen as functional. However, an etiological dimension can further explain why misrepresentations seem to be globally dysfunctional. Hence, the constructive account of meaning should be, as it seems, not only based on the current dynamics of the semiotic system but also on its (evolutionary) history.

## Mentally-ill Minds

In this section, we focus on mental disorders in non-human minds, especially obsessive-compulsive disorders and delusions. Mental impairments in animals are traditionally understood in pure behavioral terms, which we find insufficient. We will argue that it is plausible to apply the concept of mental misrepresentation to non-human minds in case of mental impairments at least in some cases, even though such applications are not without their problems.

Animal^1^ psychopathology studies mental and behavioral disorders in non-human animals (Keehn 1979). The research conducted in this approach shows that animals other than humans can be mentally ill like people. A huge limitation in this field is that the cognitive aspect of mental disorders among animals is usually ignored (Kalueff and Tuohimaa 2004), although reports on mental disorders in animals indicate that the implicit assumption in the study of cognitive processes in animals is that they represent their environment or other mental states. This tension may be still part of the Cartesian heritage in studying animal behavior: animals are explicitly held not to have any beliefs or other cognitive states (which is justified by the appeal to explanatory parsimony principles), and at the same time, their behavior seems almost impossible to explain in other terms.

The classic position in animal psychology assumed that all mental states are conscious. It is opposed to behaviorism, which is the most commonly identified with studies on animals. This classic position must, however, be weakened. That is to say, in the case of research on mental disorders in animals – and thus their erroneous representations – it is admittedly difficult to talk about the study of genuine representations. Of course, we mean representations not derived from the observer’s interpretation, because confirming hypotheses about mental representations is empirically challenging. However, the research on mental disorders in animals and the related controversial concept of suicide in non-human animals, are based on the representational hypothesis that animals represent their future. This hypothesis plays a heuristic role, and it seems that without it there would be no progress in the study of mental disorders in animals. The heuristic may be fallible but it has turned out very fruitful in other kinds of studies of cognitive process in animals, and prominently in the study on cognitive maps in rats and, in particular, on place cells in the rat (Bechtel 2016). Still, this is not to say that all mental impairment in animals is explainable by recourse to mental representation or semiotic phenomena in general. All we claim is that at least some impairments may be related to deficits of representational capacities.

Usually, mental disorders in non-human animals are regarded from an evolutionary point of view as non-adaptive behaviors caused by some sort of cognitive or emotional impairments (Braitman 2014). Historically, the list of animal psychopathologies started with depression. Martin Seligman at the University of Pennsylvania in 1967 pioneered the study of depression in the animal model of learned helplessness. These experiments and the theory of learned helplessness began as an extension of his interest in depression. In his famous experiment, dogs were separated into three groups, the control group, the group which had control over when they were being shocked, and the group without control in the same predicament. After the shocking situation, the dogs were tested in a box where they could escape shock. Only the last group, in which dogs perceived that the outcome was not related to their efforts, passively took the shock. They experienced inability to avoid a harmful situation. In that way, dogs learned to behave helplessly. Seligman argued that depression is a result of so called learned helplessness. This way he showed that mental illness such as depression isn’t unique for human beings (Seligman 1975).

Another argument in favor of the claim that animals other than humans can suffer from mental illness, was that non-human animals with ‘mental problems’ often respond well to the same antidepressant or antipsychotic drugs that work for humans (Braitman 2014). Moreover, a relatively recent study, published in *Science,* showed that not only vertebrates, but even invertebrates (like crayfish) responded to antianxiety medication. Strictly speaking, after exposure to stress, crayfish sustainably avoided the aversive illuminated arms of an aquatic plus-maze, which was correlated with an increase in brain serotonin. This behavior was abolished by the injection of the benzodiazepine anxiolytic chlordiazepoxide (Fossat et al. 2014). Although the connection to animal mental illness seems to be thin, because even in humans not only mentally ill but all humans usually respond to antidepressant or antipsychotic drugs, the case of crayfish is surprising. In this experiment invertebrates responded to anxiolytic medicine like patients with anxiety disorders: crayfish behavior was interpreted as “bold” and “combative”. Finally, it is estimated that value of the *animal* psychopharmaceutical industry in US was seven billion dollars in 2011 and nine billion dollars in 2014 (Braitman 2014).

Some other mental illness in non-human animals are categorized like those diagnosed in human beings (mood disorders, anxiety disorders, somatoform disorders etc.). Besides, there are some disorders which are not observed among humans, e.g., in eating disorders: activity anorexia, Thin sow syndrome or Pica (Dodman 2016). Despite this, in the rest of the paper, we will restrict ourselves to consider only two phenomena: delusions and obsessive compulsive disorders.

According to DSM 5 (Diagnostic and Statistical Manual of Mental Disorders Fifth Edition), delusion is defined as follows:

A false belief based on incorrect inference about external reality that is firmly sustained despite what almost everyone else believes and despite what constitutes incontrovertible and obvious proof or evidence to the contrary. The belief is not one ordinarily accepted by other members of the person's culture or subculture (e.g., it is not an article of religious faith). When a false belief involves a value judgment, it is regarded as a delusion only when the judgment is so extreme as to defy credibility (APA, 2013, p. 819)

This definition raises many questions. For our purposes, it is important to note only three main problems with this description of delusions. First, this definition seems to be too broad, it describes many types of beliefs that are not delusions. For example, the definition implies magical thinking is delusional; magical thinking is a belief that one’s thoughts by themselves can bring about effects in the world or that thinking something is the same as doing it, or kind of thinking like “When I’m passing by the lantern, I cause it to go out” (although it actually goes out when no one passes by). Second, many researchers and theoreticians strongly disagree with the claim that the delusion is a kind of belief. They emphasize that the character of delusions is different (e.g. doxastic (Bortolotti 2009); notional (Currie and Jureidini 2001), experiential (Gold and Hohwy 2000); or simply delusions are considered as empty speech acts with no intentional import (Berrios 1991)). Thirdly, it is difficult to apply the above definition of delusions to non-human animals unless we recognize that they always live in some kind of the culture. But this problem seems easy to solve by replacing “other members of the person's culture or subculture” in the definition by “other members of the same species (or population)”.

Delusions are almost exclusively diagnosed in human beings. It is probably the Cartesian heritage that animals do not have beliefs, hence cannot be delusional. Most accounts of animal mental illness are Cartesian by not allowing delusions owing to Cartesian reasons. For example, in a recent review it’s stressed that “they involve dysfunctions of what many consider uniquely human faculties like consciousness, language, reality monitoring and meta-cognition” (Corlett et al. 2010, p. 346). While a major qualifying factor of a delusion must be the presence of cognitive thoughts, in animal psychopathology such a thought aspect in delusion is simply ignored. It means that before non-human animals can be diagnosed with a mental disorder, there are no explicit assumptions about their mental processes. Representations are otherwise confirmed to exist in animals (cognitive maps in rodents, (Ivernhe et al. 2012), structural models in pigeons (Rilling and Neiworth 1991) etc.). So it seems quite implausible to suppose that no delusion can exist in animals. Note also that if we’re right, a proper diagnosis of delusions in animals might help build further animal models of delusions, which are currently extremely difficult to justify (Corlett et al. 2010). The same applies to hallucinations, which is only rarely diagnosed in dogs or cats (DeLahunta and Glass 2009).

Thus, to summarize, we propose to define delusions in animals as follows:

An inaccurate representation based on incorrect inference about external reality that is firmly sustained despite what constitutes incontrovertible and obvious proof or evidence to the contrary. The representation is not one ordinarily accepted by other members of the species (or population).

Note that we take “inference” not to require verbal capacities, and we assume that there may be inferential processes in perception; hence, under this definition, there is no in-principle distinction between delusions and perseverant hallucinations.

While cognitive or hallucinatory impairments are diagnosed only rarely, the most popular diagnosis is obsessive-compulsive disorder (OCD). In animal psychopatology, OCD occupies a special place. OCD in animals can be defined as a specific, unnecessary action, repeated more often than would normally be expected. Obsessive-compulsive animals can develop compulsions in that they are engaged a whole day. So they don’t eat, drink or go for a walk but repeat their compulsive moves. Those fixations like shadow-chasing, chasing their tails or licking paws compulsively are characterized by doing exactly the same thing in exactly the same order. Such compulsive behaviors, such as the most famous case of a polar bear named Gus in Central Park Zoo, who would swim figure eights for 80% of his waking hours (Grandin and Johnson 2010), can keep animals calm. In the wild, polar bears swim for miles but in captivity, they compensate the lack of natural activity by demonstrating stereotypies, i.e., constant repetitive behavior patterns that do not have an apparent purpose or function. That’s why it was difficult to prevent Gus from exhibiting repetitive swim in the zoo. Only after zookeepers provided him barrels in his pool which he could play with, Gus engaged in compulsive swimming 10% of the walking hours and was able to eat, drink and eventually returned to the physical health. There are studies showing that particularly captive animals participate in compulsive behaviors (Dodman 2016).

It seems reasonable to regard compulsive behaviors as a way to reduce anxiety that correlates with intrusive and often preposterous thoughts called ‘obsessive thoughts’. So only when an obsessive thinking is stopped by a kind of distractor, like a barrel, an animal can start to behave ‘normally’. Qualifying factor of OCD as the presence of such obsessive thoughts, where compulsions are exactly a method of reducing anxiety caused by obsessions, is problematic (Szechtman et al. 2016). As many researchers claim, we can’t know the motivation for compulsive acts in animals. The problem whether animals are able to ‘obsess’ is sometimes resolved through replacing the term OCD with a less misleading concept of ‘abnormal repetitive behavior’ (Holden and Travis 2010). However, this term might be equally misleading as it does not reveal that there might be a representational component in the disorder.

It’s worth noticing that it remains a challenge to construct either theoretical models or empirical experiments that could show clearly whether some OCDs or other psychopathologies have a representational character. The problem lies, of course, in the complex nature of various kinds of psychopathologies, so concentrating on one cognitive aspect is insufficient. Furthermore, the research methodology in animal psychology and psychiatry is still in its infancy. The lack of verbal communication with animals makes investigation much more difficult. It is, however, plausible to expect that the complex methodology used to study cognitive maps in animals could be useful in this context.

Let us conclude this section. In animal psychology, instead of delusions or similar cognitive mental disorders, OCDs are being diagnosed, even if it’s plausible to hypothesize that they both have a representational or semiotic component. While in other fields of animal psychology and neuroscience, representations have already been confirmed to exist quite well, animal psychiatry still seems to shrug at the very thought. We will however assume that at least some OCD symptoms are linked with delusions or other representational phenomena. Let us see how this fits the constructive framework in biosemiotics then. We will propose our account but in order to do that, firstly we shall focus on Bickhard’s account of mental representation and function as well as Millikan’s etiological function. They both are biologically plausible, and thus helpful in explaining mental misrepresentations in non-human minds.

## Misrepresentation as Cognitive Malfunction

In this section, we show how an organism can have mental misrepresentations. We focus on functional theories of representations that include misrepresentation understood in terms of dysfunction. Such accounts differ from traditional accounts of mental representations, e.g., simple causal theories, in which there is no place for dysfunction and misrepresentations are simply not functional at all (for an elucidation why such causal theories have problem with misrepresentation, see Bielecka 2016). Furthermore, the capacity of a cognitive system for misrepresentation can help account for (limited and evolutionarily merely satisficing) rationality of biological agents. First of all, no rational biological agent is faultless. And only cognitive agents capable of misrepresentation actually also represent. Second, cognitive agents may commit mistakes that would render them irrational unless we could explain their behavior as caused by their misrepresentation. For example, someone may sit on the bus stop even if no bus is coming. If we know that the bus no longer stops there, sitting there in order to get somewhere is irrational; however, it may be explained as rationally justified (and caused) by someone’s misrepresentation of the bus schedule. Hence, someone at the bus stop may simply be in error about the bus schedule; she is not faulty of trying to get transported to the city center by just sitting on the bench. This shows that it’s not global irrationality that is the main problem with the person on the bus stop; it’s the local misrepresentation. Preserving the assumption of her rationality without some form of misrepresentation would be difficult if not completely implausible. We take the possibility of system-detectable error or *misrepresentation for the system* to be a necessary condition for the satisfactory account of mental representation in biological agents.

First, we will follow Mark Bickhard to show how misrepresentations are possible as dysfunctional representations. In order to do that, first, we introduce Bickhard’s account of mental representation and his account of function. Then, we show that his account of function needs an etiological dimension in order to better explain why representational mechanism can be functional and further how mental disorders can be partly dysfunctional. That’s why in our account of representation, we apply a hybrid account of function, conjoining Bickhard”s and Millikan’s accounts of function.

Bickhard understands mental representations in terms of his interactivist model. According to his model, all organisms are interactive systems as they are self-maintenant but also they are autonomous in the sense of self-reconstruction: organisms (every organism is an interactive system) are able to “recruit and even manipulate (themselves in) their environment so as to (contribute to) maintain(ing)” (Bickhard 2009, p. 555). A representational mechanism appears at a second level of interactive systems, in a system that not only interacts with environment but also can differentiate organizational and processing properties of the first level. Only those organisms that have representational mechanisms can be fully autonomous in a sense of manipulating themselves in their environment so they are in a constant process of constructing and reconstructing their representations and confronting them with environment. Although Bickhard has an account of what a representational mechanism is, his view is not entirely satisfactory because it lacks further explanation why representational mechanisms could be adaptive.

In Bickhard’s account, *function* is accounted for in biological and dynamic (thermodynamic) terms:

Far from equilibrium processes require maintenance in order to be stable, and such maintenance is functional relative to the stability of that system—it serves a function insofar as it contributes to that stability (Bickhard 2009, p. 555).

In this view, a feature X is functional only if X helps a system to self-maintain itself as a system far from its thermodynamic equilibrium. Basic representations (emergent from the basic structure of the biological organism) are functional because they play a current role in its self-maintenance while indicating organism’s future possible actions (a similar account of intentionality is defended by Deacon 2012). The representational function helps an organism to stay far from its thermodynamic equilibrium; thanks to such a function, the organism can anticipate its action results, as the representational function is related to anticipations of action results, and the organism can survive or even learn something. These anticipations of future possible actions have satisfaction conditions. An organism or its subsystem can detect whether these are satisfied by confronting anticipations with results of actions caused by them. Furthermore, the idea that organisms themselves recognize the adequacy of their representations is enshrined in Bickhard’s notion of *system-detectable error.* So, only an organism that can detect its own errors can really anticipate its future actions. In this sense, error detectability makes representations necessary in a stability-maintenance process.

Let us illustrate this idea with a deliberately simplistic example of a frog’s mistakenly snapping a bumblebee. A frog that anticipates snapping a fly but snaps a bumblebee recognizes it as a misrepresentation while realizes that it snapped a bumblebee (as long as snapping a bumblebee has different consequences than snapping a fly; for example, it may be sour for the frog; the details here are fictional and are meant here for illustrative purposes only).
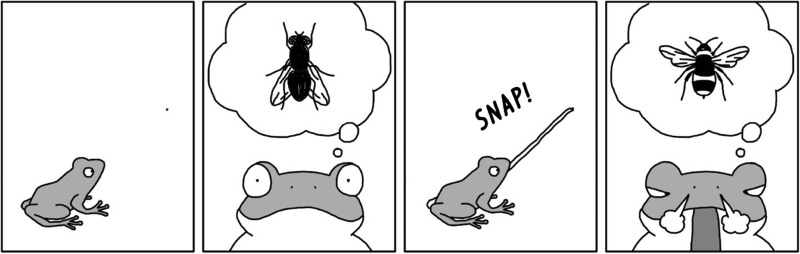


Furthermore, a frog can use its own error in a process of learning that in particular conditions, the representation of an object X as a fly, is a misrepresentation, because it leads to wrong actions. However, Bickhard’s approach seems to be somewhat unsatisfactory in situations in which an organism’s action fails, even though its representation is correct. For example, the frog’s representation may be correct but during snapping, the insect is eaten by a bird that flies nearby. Even if Bickhard insists that the contents of basic representations is implicit and the satisfaction conditions of actions are identical to satisfaction conditions of representations, which would mean that he could reply that the frog implicitly represented the situation that included non-intervention of birds in snapping, such an insistence is not biologically plausible. This would imply that implicit representations are unboundedly precise in representing all possible conditions of accuracy, which is what Bickhard obviously and plausibly denies. He could only reply that the frog will, in such situations, detect misrepresentation even if there is none. However, the frog’s needs are better served if it ignores such instances and detects no misrepresentation, and continues attempts of snapping if another fly appears. How would this be possible then?

To make such instances possible, in our account the criterion of correctness is not the success of action but the internal consistency of representation. Two bits of (structural) information should be compared by a system to check their consistency. If there is a problem, it may mean that one of them is in error. For example, if one is more reliable for a system, it may be inferred that this one is correct, and another will be rejected. Deacon 2012 seems to hint at a similar solution to this problem:

The consistency (redundancy) and inconsistency (non-redundancy) of the evidence is not itself a guarantee that a given interpretation is accurate. Faced with the problem of comparing alternative interpretations of the same events, one is often forced to analyze other features of the source of the information to determine if there are systematic biases that might be introducing spurious or intentionally skewed levels of redundancy. Creating the false appearance of independent sources of information is, for example, a major tool employed in propaganda and confidence schemes (Deacon 2012, p. 406).

Note however that even if Deacon uses the notion of interpretation in the above citation, the account defended here uses the notion of structural, and not semantic information, so the notion of interpretation is not required. So, if a frog has auditory information of a fly and visual information of a bumblebee and assuming that a visual information is more reliable, only then a frog has a misrepresentation of bumblebee as a fly (here also the details are meant for illustrative purposes only).

To sum up, according to Bickhard’s account of representation, the representational content is constructed by an organism in a constant process of constructing and reconstructing its representations and confronting them with its environment. An organism, or its subsystems (at least sometimes) recognizes its own errors and can correct them. However, Bickhard’s account has some problems concerning failed actions as consequences of accurate representations, and this is why we propose to supplant his account with internal consistency checking as offering a more basic mechanism for error detection. After all, recognizing that an anticipation deviates from reality also arguably requires consistency checking (between the anticipation and the detected result of the action).

In addition, the etiological dimension can make a mechanism responsible for a given type of mental representation if the mechanism appeared at least once in a history of an organism and played its role that had helped an organism to survive. According to Millikan’s (1984) biological account of function, representations are functional only if they are products of adaptive mechanisms. To have a function:

an item must also come from a lineage that has survived due to a correlation between traits that distinguish it and the effects that are “functions” of these traits, keeping in mind that a correlation is defined by contrasting positive with negative instances. Intuitively, these traits have been selected for reproduction over actual competitors. Because the correlation must be a result of a causal effect of the trait, the trait will not merely have been “selected” but will have been “selected for” (Sober 1984). Thus a thing's proper functions are akin, intuitively, to what it does by design, or on purpose, rather than accidentally (Millikan 1984, p. 8).

So an organism has a representational function F if a certain property P was selected for F. It means that such a property is positively correlated with realization of that function and it contributes to further replication of P because of the effect that P was naturally selected. Sober distinguishes two different concepts, selection *for* and selection *of* (Sober 1984, 1993). According to him, selection *for* a property P means that having that P causes success in survival and reproduction. Selection *for* is contrasted with selection *of*; it says why some properties increased in frequency while the former one describes causes, the latter–effects of selection:

to say that there is *selection for* one trait (Fast) and against another (Slow) is to make a claim about how those traits causally contribute to the organism's survival and reproductive success (...) One trait may be fitter than another because it confers an advantage or because it is correlated with other traits that do so. (Sober 1993, p. 83)

A trait of having a heart by an organism is adaptive for pumping blood in a population when their members have hearts as a result of earlier selection for having a heart and also because having a heart contributed to their fitness because the heart pumped blood. So, having a heart was selected *for* in a sense it has a role in biological adaptation, because it helped an organism to survive while pumping blood, but the heart-beat was selected *of* in the sense that it is a latter-effect of selection, not causally important for a heart to function properly. Applying Sober’s terminology to representational mechanisms, while a representational mechanism was selected *for* in the sense that it has a role in biological adaptation, vehicular property are selected *of* in a sense it is an effect of selection.

According to Bickhard and Christensen, what makes the etiological account of function different from the dynamical one is that only the latter emphasizes serving a function, which they find more important to understand as it is actually much more important in the system dynamics:

In certain respects we are simply focusing on a different issue: etiological theory takes as its task the problem of explaining what it is for a part of a system to have a function, whereas we focus on what it is to be an adaptive system, and on the nature of serving a function relative to such a system (Christensen and Bickhard 2002, p. 4)

Moreover, Bickhard and Christensen notice some problems with the etiological account of function and argue that etiological function is epiphenomenal. Let us introduce an iconic thought experiment used in a discussion on causal relevance of mental representations (Davidson 1987; Christensen and Bickhard 2002; Bickhard 2009; Krohs 2007). Imagine a lion that springs into existence and is atom-by-atom identical to a real lion.^2^

According to Millikan, only the real one has functions because it has evolutionary history, while the science-fiction lion doesn’t. Bickhard and Christensen find her claim counterintuitive:

Function, in this view, is dynamically—causally—epiphenomenal. It makes no difference to the causal or dynamic properties of an organism whether or not its organs have functions. Etiological models thus fail to naturalize function. Etiological history explains the etiology of something, but it does not constitute any of the causal or dynamic properties of that something. Etiology cannot constitute the dynamics of what it is the etiology of (Bickhard 2009, p. 558)

So history doesn’t constitute any causally-relevant property – it is causally epiphenomenal. Although such a consequence seems to be intuitive in an artificial example of a science-fiction lion, it doesn’t in biological ones. In our view, a representational mechanism seems to be still functional even though it doesn’t work properly because it produces delusions and they fail to be corrected because the system is no longer capable of detecting error in some of its representations. But this mechanism normally has a feature that allows it to correct error; this feature has been selected for detecting error. However, this feature currently has no effect, or is causally epiphenomenal in the system, and does not serve its function anymore.

We propose, in essence, to assume a hybrid account of function (Davies 2001) that includes both etiological and dynamical dimensions. The notion of etiological function is used to account for the why-questions concerning the biological structures, while the dynamical function is best understood as answering the how-questions that pertain to the current dynamics. Therefore, both can be used in a more complex, multidimensional account of biological functionality, which has its roots in Tinbergen's (1963) account of explanations of behavior (Miłkowski 2016).

In this account, cognitive errors themselves are currently dysfunctional (as in dynamical function) but the representational mechanism is adaptive, or etiologically functional. Hence, this mechanism has also a function to detect its own errors, even if it is not currently performed or served. Surely Bickhard doesn’t claim that error should be always detected so he would admit that an error detection mechanism is dysfunctional, but it isn’t possible for him to claim that it is an error detection mechanism if its damage is *persistent* in an organism. If such a damage is inborn, there is no evidence that such a mechanism has a role to detect errors because it has never played such a role in an organism so it didn’t contribute even once to being far from dynamical equilibrium. In contrast, according to Millikan’s account of proper function, this mechanism can be classified as having a proper function that is now not served. That’s why supplying Bickhard’s account of function with etiological elements helps to save the basic principle of his account.

## Misrepresentations in Non-human Minds

In this section, we will extrapolate Bickhard’s account of system detectable-error to other cognitive impairments present in mentally-ill minds. We will argue that such impairments can be understood in terms of mental misrepresentations in Bickhard’s model. We argue that Bickhard’s account of function, amended by adding a historic dimension to it, can account for misrepresentation.

Misrepresentations may also play a role in an organism’s self-maintenance because they help to preserve a cognitive system’s organizational structure. In this sense, they serve a function in the system, at least in Bickhard’s account of function. Here, however, lies a problem with understanding the role of cognitive impairments in Bickhard’s account. Cognitive impairments are, in his view, functional as far as they help an organism to preserve its organizational structure. His dynamic account of function fits OCDs very well. But OCDs are diagnosed as disorders, which means that they should be in some way, even if derivatively, biologically dysfunctional.

One solution to this problem would be to claim that stereotyped behaviors are not in general functional as they make the biological agent less flexible in its interactions with the environment, which, in turn, do not help maintain the agent’s autonomy. But this would be too quick. Contrary to this line of argument, some stereotyped behaviors among animals can be beneficial. They are known as ‘fixed action patterns’ although firstly were considered as pathological or maladaptive ritualized actions (Kalueff et al. 2007). The problem of dysfunctionality of some stereotyped behaviors cannot be, hence, solved in such a simple way. Therefore, we’ll return to this thread after etiological concept of function been introduced.

Another problem is that in some disorders, misrepresentations seem to be at their root. For example, delusions are clearly misrepresentational. But a mentally-ill mind may be unable to detect misrepresentation anymore. This is what delusions are; they may go undetected and are not, normally, corrected by deluded organisms. One of the influential neuropsychological accounts of monothematic delusion claims that they should be explained as caused by two factors: one responsible for its production, and the second responsible for its permanence, or the failure to properly evaluate delusional representations (Coltheart et al. 2011). But the failure of proper evaluation has dramatic consequences for the constructive account of representation under discussion. According to Bickhard’s account of representation, a system or its subsystem must be able to detect misrepresentation in its representations at least sometimes to be able to represent at all. So delusions are strictly *impossible* under the assumptions of this constructive approach to meaning. And just because they may, at least sometimes, help maintain the organism’s autonomy, they would be treated as functional. However, these representations are totally out of touch with reality. Would this mean that constructivism embraces the view that there is no meaningful distinction between delusions and other representations?

Enriching the dynamical account of function with historicity or etiology, as proposed in Section 2 above, seems to be a solution to the problem. It would be a mistake to conclude that the whole representational mechanism is no longer representational, and that delusions are not representations. It has been selected for its representational feature but under current circumstances, it does not serve its function completely: it produces representations but is unable to correct some of them.

According to this hybrid account of function, delusions are representational phenomena that persist due to the fact that they would have been corrected, were the mechanism of error detection still functioning. Hence, the hybrid account can preserve delusions and obsessive thoughts in OCDs as partially dysfunctional. Delusions are representations but their persistence is not functional, in contrast to what a system-based account of function implies. Putting it differently, they are dysfunctional due to the failure of recognition mechanism, so they are not adaptive.

Note that the same solution may be applied to OCDs as well. Even if they are in some respect beneficial for the self-maintenance of a given organism, they are malfunctions because neither mechanisms producing OCDs, nor abnormal repetitive behaviors were selected for because of producing OCDs or abnormal behaviors by the natural selection. So instead claiming that stereotyped behaviors are all dysfunctions, we can claim that these that are caused by malfunctions in subsystems that were selected for indeed are dysfunctional.

This is related strongly to our argument of rationality, saying that 1) only cognitive agents capable of misrepresentation (such as delusions or OCDs) actually represent and also 2) mistakes done by cognitive agents render them irrational unless explained their behavior as caused by their misrepresentation.

Thus, we conclude that the constructive approach to meaning can be defended only when accompanied by a hybrid account of biological functionality. Otherwise, it does not seem to be suitable for describing permanent representational malfunctions that make system-detectable error impossible (due to a biological disorder).

## Interim Summary

Before we summarize, let us take stock. We will illustrate the discussed accounts of misrepresentation and function with deliberately idealized examples of OCDs and delusions.

Let us explore an example of a Gus the bear, living in the zoo and making circles in a way of eights as a sign that it cannot escape from the zoo. Assuming that such an action is driven by the bear’s mental representations, a bear then misrepresents his action as a leading to an escape from his predicament whereas it obviously does not contribute to escaping the zoo. It seems quite plausible to classify Gus’s sequential behavior as a fixed action pattern that is beneficial for the animal, being locally functional. Obviously, some reservations concerning the plausibility of this scenario would be in place. According to Bickhard’s account of function, the representational function helps Gus in stability-maintenance process and in his interactivist model, Gus is able to detect its own errors by confronting anticipations of escape with results caused by such anticipations. Quite obviously, something goes wrong and he doesn’t detect the error (or at least doesn’t learn anything from the error).

Adding a criterion of consistency, we assume that the bear is able to detect an error between bits of information. It can be the case that bits of information come at different timescales, information A of being on the way out and further information B of being in the zoo. Although the representation of escape is not satisfied (and thus inaccurate) for the bear, it can’t stop repeating the same type of action, so can’t correct its misrepresentation. Bickhard’s account of function allows us to consider such fixed action patterns caused by a representational mechanism as locally functional, because they help the bear to maintain its existence. However, to understand why misrepresentations of escape do not lead to real escape and are globally dysfunctional, we need to add an etiological dimension to Bickhard’s account of function. The etiological function can explain why Gus’s representational mechanism remains functional although representations of that type are currently dysfunctional - they are no longer adaptive. That’s why a hybrid account of function that conjoins the dynamic and etiological account of function can deeper explain partial dysfunctionality of OCDs and functionality of the representational mechanism at the same time.

Let us consider a delusion example, say a dog constantly hallucinating a fly. Delusions are more obvious examples of misrepresentations, because the dog represents a fictional fly. In Bickhard’s interactivist model the dog is able to detect its own errors by confronting anticipations of a fly with results caused by such anticipations that a fly is actually a piece of air. The dog can test it in action, for example by tactile senses of a tongue catching a fly. However, if the delusion of the fly persists, it cannot be easily accounted for in the original interactivist model because the function to detect the error may be completely lost. Adding a criterion of consistency to the model, we can assume that the dog is able to detect an error between bits of information, as between information or representation of a fly coming from a visual or aural apparatus and information coming from the tactile apparatus. In order to understand better why delusions are dysfunctional, we add an etiological dimension to that, which helps us to consider delusions as maladaptive. So a hybrid account of function is much more fruitful than either dynamic or etiological function considered separately. Understanding psychiatric disorders as misrepresentations by conjoining Bickhard model with criterion of consistency as well as putting together dynamic and etiological accounts of function seems to be quite promising as a scientific program, even if going beyond simplified examples presented herein will require gathering substantial amounts of empirical evidence.

## Conclusions

The aim of our paper was to argue for a constructive approach towards mental misrepresentation in non-human psychopathology. We used Bickhard’s account of mental representation (system detectable-error), according to which all meaning is created autonomously in an organism. We tried to show that some mental impairments are better considered as involving complex animal representation and misrepresentation. To do that, we considered the idea of misrepresentation for non-human animals in Bickhard’s model with some amendments. In such a model, mental impairment is not seen as a symptom of full-blown global irrationality, and such impairments may occur in humans and animals. Some disorders may be accompanied by misrepresentations, and even if they are currently misdiagnosed or underdiagnosed in animals, due to excessive caution, the constructive account of meaning is well equipped to account for it as it has resources to deal with misrepresentational phenomena.

We argued however for considering inconsistency between different bits of information as the symptom of representational inadequacy, which can then be used to detect the error. We also showed that in order to consider psychiatric disorders representational we have to add to Bickhard’s dynamic account of function an etiological dimension. Without the consistency criterion a cognitive system wouldn’t be able to recognize its misrepresentation, such as delusions, in order to possibly correct them, which is the core of the system’s rationality. According to Bickhard’s dynamic account of function, misrepresentations, such as delusions, are functional because a mechanism of error detection still functions. An etiological dimension further explains why they are partly functional in rational system, because a whole representational mechanism was selected for organism’s fitness and rational behaviors manifested in detecting and correcting mistakes are useful. But particular types and tokens of misrepresentation are dysfunctional because they are no more adaptive.

We sketched how to apply this framework to OCDs and delusions. Therefore, it is necessary to suggest some perspectives for further work. In particular, it would be important to see whether our model of mental misrepresentations is compatible with other types of mental disorders. Especially interesting class are mood disorders, in particular depression, because of the relationship between mental and affective component. We strongly believe that an evolutionary approach to cognition that justifies a connection between animal and human cognition can show new paths in research in psychiatry, which, first, can help psychiatrists to understand better both animal and human disorders, and second, can help to improve the treatment of mentally-ill animals.

## Footnotes

1 In the paper by “animals” we mean other mammals.

2 The example above has been discussed in this form in a number of influential papers even if it is utterly unrealistic but the idea of such a thought experiment can be paraphrased in Sober’s language as follows: A was selected for B that plays no role any more, so it couldn’t be selected for B. B in this case is an exaptation. In Millikan’s example (Millikan, 2002), an otter’s stomach covered by fat is now used as a table in the sea, but a stomach was not selected for being a table.
